# New N-Methylimidazole-Functionalized Chitosan Derivatives: Hemocompatibility and Antibacterial Properties

**DOI:** 10.3390/biomimetics8030302

**Published:** 2023-07-11

**Authors:** Natalia Drozd, Alexey Lunkov, Balzhima Shagdarova, Alla Il’ina, Valery Varlamov

**Affiliations:** 1 National Medical Center for Hematology, 4, Novoi Zykovsky Prospect, Moscow 125167, Russia; 2 Institute of Bioengineering, Research Center of Biotechnology of the Russian Academy of Sciences, Leninsky Prospect, 33, Build. 2, Moscow 119071, Russia; fwnf1994@gmail.com (A.L.); shagdarova.bal@gmail.com (B.S.); ilyina@biengi.ac.ru (A.I.); varlamov@biengi.ac.ru (V.V.)

**Keywords:** chitosan, chitosan derivatives, click chemistry, hemocompatibility

## Abstract

Novel imidazole derivatives of the low molecular weight chitosan N-(2-hydroxypropyl)-1H-1,2,3-triazol-4-yl)methyl)-1-methyl-1H-imidazol-3-ium chitosan chloride (NMIC) were synthesized using copper-catalyzed azide–alkyne cycloaddition (CuAAC). The degrees of substitution (DSs) for the new derivatives were 18–76%. All chitosan derivatives (2000 µg/mL) were completely soluble in water. The antimicrobial activity of the new compounds against *E. coli* and *S. epidermidis* was studied. The effect of chitosan derivatives on blood and its components was studied. NMIC samples (DS 34–76%) at a concentration <10 μg/mL had no effect on blood and plasma coagulation. Chitosan derivatives (DS 18–76%) at concentrations of ≥83 μg/mL in blood and ≥116.3 μg/mL in plasma resulted in a prolongation of the clotting time of blood and plasma, positively related to the DS. At concentrations up to 9.1 μg/mL, NMIC did not independently provoke platelet aggregation. The degree of erythrocyte hemolysis upon contact with NMIC samples (2.5–2500 μg/mL) was below 4%. The inhibition of blood/plasma coagulation indicates the promising use of the studied samples to modify the surface of medical materials in order to achieve thromboresistance.

## 1. Introduction

Chitosan is a heteropolysaccharide consisting of β-1,4 linked units of D-glucosamine and N-acetyl-D-glucosamine, which are randomly distributed in the polymer chain. Chitosan is a partially deacetylated form of chitin. Chitosan is a cationic biopolymer by nature, and it is this factor that determines its functionality. The physicochemical characteristics of chitosan, such as the molecular weight, degree of deacetylation and distribution of acetyl groups in the biopolymer chain, affect its biological properties. The presence of a hydroxyl at the C3 and C6 atoms and amino and acetamide groups at the C2 atom determines the physicochemical properties and biological activity of chitosan. Chitosan has antimicrobial and chelating activities and useful biomedical properties—biocompatibility, biodegradability and low toxicity [1,2]. Chemical modification is a way to improve the properties of chitosan. Quaternary chitosan derivatives are characterized by an increased solubility and antimicrobial properties [3]. They can form complexes with anionic polysaccharides, including heparin [4].

Click chemistry is a universal tool for the chemical modification of chitosan. Recently, many researchers [5,6,7,8] have applied CuAAC in chitosan chemistry. Mild reaction conditions contribute to the preservation of the starting material characteristics. In previous work, we synthesized new quaternary derivatives of chitosan containing a trimethylammonium moiety using click chemistry [9]. Introduction of heterocyclic compounds into the structure of quaternized chitosan can increase the antimicrobial activity of the compound. Previous chemical modifications of chitosan with heterocycles have shown promising biological activities [10,11,12,13]. In a number of works, the use of chitosan derivatives, including imidazole derivatives, has been shown in the development of hemocompatible delivery systems for medicinal products or to achieve the thromboresistance of surfaces of medical devices/materials (nanoparticles, frameworks, hydrogels and membranes) in contact with blood [14,15,16,17,18].

Heterocycles may be part of the emerging protective nanoscale coating that appears on the surface during experimental modeling of the interaction of a heart valve made of carbon pyroceramics with human blood plasma [19]. The film is formed as a result of the release of anions of the corresponding α-amino acids (amino acid residues of complex chains of blood proteins) containing heterocyclic rings. The structure of the organic heterocyclic compound imidazole is represented by a five-membered ring with two nitrogen atoms and three carbon atoms in the ring. Imidazole was previously used in new materials, e.g., metal–organic-framework-incorporated biocomposites. Researchers [20] investigated a self-assembled material consisting of a chitosan-based membrane loaded with a copper boron imidazolate scaffold as a wound dressing for the treatment of a wound infected with bacteria.

There is great interest in such biopolymer polysaccharides as cellulose, chitosan, starch, chitin and alginate to create natural substitutes for synthetic polymers [1]. Chitin and its derivatives find practical applications in chemistry, agriculture, medicine and cosmetics, as well as in the textile and paper industry. Recently, chitosan has also aroused great interest, including in the field of biomedicine. Chitosan is a biopolymer derived from chitin, which has demonstrated great potential for use in tissue regeneration and controlled drug delivery [14]. Medical devices/materials in contact with blood are widely used in the diagnosis and treatment of various diseases [21].

Materials used in medical devices that are in contact with blood must have biocompatibility, hemocompatibility, thrombosis resistance and appropriate mechanical properties. For the manufacture of such devices, different materials are used, e.g., metals, alloys, ceramics, carbon materials, polymers, etc. [22,23]. However, in some cases (intravascular stents as well as non-implantable catheters), thrombus formation is still a problem [24]. Surface modification is an effective strategy to give biomaterials specific surface functions, including biocompatibility. Coating the surfaces of biomaterials improves their biocompatibility, thus avoiding side effects. Both inorganic materials and polymers can be used for surface coatings. For these purposes, synthetic polymers are most often used in biomedicine, such as polylactic acid, poly(lactide-co-glycolic) acid, poly(ε-caprolactone) [25], polyurethane (PU) [26] and biopolymers such as cellulose [27] and chitosan [28].

Previously, we modified silicone layer-by-layer (LbL) using chitosan and unfractionated heparin (UFH), and quaternized chitosan and UFH. The silicone was activated with cold oxygen plasma. Modification of silicone led to an increase in its resistance to the formation of blood clots [29]. Modification of isocyanate-activated PU by the LbL method using chitosan and UFH also led to an increase in the clotting time and improved the thromboresistance of the material [30]. The synthesis of new chitosan derivatives with hemocompatible and antibacterial properties is an urgent task, since such compounds can be used in the future both on their own and as part of devices when modifying their surface.

The aim of the work was the synthesis of new promising cationic derivatives of chitosan using click chemistry and an investigation of their physicochemical properties and hemocompatibility to determine the influence of new derivatives on the plasma and cellular components of human blood in experiments in vitro.

## 2. Materials and Methods

### 2.1. Chemicals and Reagents

Epichlorohydrin, sodium azide, propargyl bromide, paraformaldehyde, ammonium chloride and sodium chloride (Sigma, New York, NY, USA) were used for synthesis. Dialysis tubing, MWCO 12,000–14,000 from regenerated cellulose (RC), diameter 16 mm (SERVA LLC, Heidelberg, Germany), was used to remove byproducts.

### 2.2. Preparation of Low Molecular Weight (LMW) Chitosan

High molecular weight crab chitosan (LLC Bioprogress, Shchelkovo, Russian Federation) with a molecular weight (Mw) of 1040 kDa and a degree of deacetylation (DD) of 85% was used. It was hydrolyzed with nitric acid to obtain a fraction of LMW chitosan, Mw 47 kDa, DD 93% [31], on the basis of which all derivatives were synthesized.

### 2.3. Characterization of Chitosan and Its Derivatives

The Mw of chitosan was determined by high performance liquid chromatography using a GPC PSS NOVEMA Max analytical 1000 A column (PSS, Mainz, Germany). An elution rate of 1.0 mL/min, 0.1 M NH4, and acetate buffer + 0.2 M NaCl at 30 °C, pH = 4.5 were used. The control and analysis of chromatograms were carried out using “Multikhrom” version 1.6 software (LLC Ampersand, Moscow, Russian Federation). Pullulans (M_w_ = 342, 1260, 6600, 9900, 23,000, 48,800, 113,000, 200,000, 348,000 and 805,000 Da) (PSS, Germany) were used as calibration standards. The 1H NMR spectra of chitosan and its derivatives were recorded on a Bruker Avance 700/Bruker Avance III 600 spectrometer (Bruker, San Jose, CA, USA) operating at a frequency of 700/600 MHz at 25–50 °C.

### 2.4. Synthesis of 2-(Azidomethyl)oxirane

The procedure was performed as in previous work [9]. The spectral data of the final and intermediate products were in agreement with those previously reported [32].

### 2.5. Synthesis of N-(3-Azido-2-hydroxypropyl) Chitosan (AzCH)

The procedure was performed as previously described, by addition of different amounts (0.25–3 mol./mol. of chitosan) of 2-(azidomethyl)oxirane to chitosan at 60 °C [9]. The DS for the corresponding AzCH1-5 was 18–76%. The DS of derivatives was determined by integration of anomeric proton signals of the 1H NMR spectra, using the following equation:

$$DS\left(\%\right)=\frac{H1}{H1+H1^{\prime }+H1\prime \prime }\times 100\%=\frac{H1}{H1+H1\prime +\frac{1}{3}HAc}\times 100\%$$

where H1/H1′/H1″ are signals of anomeric protons of substituted, unsubstituted and acetylated units in the polymer chain.

### 2.6. Synthesis of 1-Propargyl-3-methylimidazolium Bromide

To the solution of 1-methylimidazole (4 mL; 51.2 mmol) in acetone (20 mL), propargyl bromide (4.8 mL; 63.2 mmol) was added. The reaction mixture was stirred at RT for 30 min, then at 50 °C for 3 h. The next day, the upper layer was decanted and the product was washed a few times with acetone. The reaction mixture was evaporated on a rotor to obtain 9.7 g of viscous yellowish oil (94%).

1H NMR (700 MHz, D_2_O): δ 7.47 (s, 1H), 7.46 (s, 1H), 4.96 (s, 2H), 3.8(s, 3H), 3.69 (s, 1H); HRMS (MnESI) m/z calculated for C_6_H_8_N_2_^+^ 121.0761, found 121.0760.

### 2.7. Synthesis of 1-Methyl-1H-imidazol-3-ium Chitosan Derivatives (NMIC)

The chitosan derivative with an N_3_ group (100 mg) was dissolved in 6 mL of 1% AcOH. Copper turnings (0.2 g) and 1-propargyl-3-methylimidazolium bromide (20% molar excess) were added into the reaction flask. The reaction mass was treated in an ultrasonic bath for 20 min with 100 W at 35 kHz. The next day, the reaction product was filtered, precipitated with acetone and washed with ethanol. Then, the reaction mass was dialyzed against NaCl for 24 h, and then distilled water. The DS of the derivatives obtained corresponded to the DS for the corresponding AzCH.

### 2.8. Solubility of NMIC Derivatives

The solubility was determined for the pH range of 3.0–12.0 by the method of turbidimetric titration. NMIC (10 mg) was dissolved in 5 mL of 1% acetic acid, and the solution was gradually titrated with NaOH (2 M) to the final pH with constant stirring. The obtained values were recorded using an HI 8314 pH meter (Hanna Instrument, Smithfield, RI, USA). Absorbance measurements of the solutions were taken at λ = 600 nm in quartz cuvettes (1 cm) on a Specol spectrophotometer (Analytik Jena, Germany) [33].

### 2.9. Antibacterial Activity of NMIC Derivatives

*Escherichia coli* ATCC 25922 and *Staphylococcus epidermidis* 33 GISK strains were used to determine the antibacterial activity. The cultures were stored in LA medium at 4 °C. An inoculum was prepared by transferring one bacterial colony in 20 mL of LB medium (18 h at 37 °C, 150 rpm). Serial double dilutions of chitosan and its derivatives were prepared in a 96-well round bottom plate. Subsequently, a suspension of bacteria in nutrient medium was added to the solutions (microbial load was 10^5^ cells/mL). The sterile nutrient medium served as a medium purity control and the suspension of test cultures in liquid nutrient medium without drugs was the growth control. The experiment was continued for 24 h at 37 °C. To assess the results, the growth of the culture in the presence of the drug was visually compared with a ‘negative control’ containing the inoculum. The minimum concentration of the substance that completely inhibited the growth of the culture was defined as the minimum inhibitory concentration (MIC) [34]. Each assessment was performed at least three times to ensure the reproducibility of results.

### 2.10. In Vitro Hemocompatibility of NMIC Derivatives

The study used blood stabilized with a solution of 0.106 M Na_3_C_6_H_6_O_7_. Blood was taken in a plastic syringe S-Monovette 5 mL 9NC (Sarstedt, Germany) from the ulnar vein of donors (all donors gave written informed consent to the collection and use of blood at the National Medical Research Center for Hematology, Moscow, Russian Federation). To obtain platelet-rich human plasma, blood was centrifuged for 7 min at 1000 rpm and at room temperature. Platelet-depleted plasma was obtained by centrifugation of blood for 20 min at 3000 rpm. Tris-HCl buffer (0.05 M) with 0.175 M NaCl, pH 7.4, was used to dissolve NMIC derivatives. The study protocols were approved by the National Research Center for Hematology (Moscow, Russian Federation; State assignment No. 056-00111-21-00 dated 23 December 2020, NIOKTR AAAA-A21-121011290079-5 dated 12 January 2021). The time of the appearance of a blood clot or plasma in the tests of blood recalcification time (BRT) and activated partial thromboplastin time (APTT) was recorded on a programmable semi-automatic coagulometer APG2-01 (Minilab-701-M, NPO Emko, Russian Federation).

To assess the effect on the BRT [35], 0.1 mL of citrate blood containing NMIC derivatives at concentrations of 0.083–830 µg/mL was added to 0.1 mL of a 0.02 M calcium chloride solution (Sigma-Aldrich, St. Louis, MI, USA) and the time of clot appearance was recorded using a coagulometer.

The effect of NMIC derivatives on the plasma coagulation time in an APTT test was evaluated in accordance with the description of the method in the publication Stuart and Michel [36] and with instructions for the PG-7/1 set (NPO Renam, Moscow, Russia Federation). An amount of 0.1 mL of a mixture of ellagic acid and phospholipids was added to 0.1 mL of platelet-poor human plasma containing NMIC derivatives at concentrations of 1.163–1162.8 µg/mL. After 3 min of incubation at 37 °C, 0.1 mL of 0.025 M CaCl_2_ solution was added and the time of clot appearance on the coagulometer was recorded.

According to the graph of the dependence of the plasma coagulation time on the concentration of NMIC derivatives, concentrations of 2APTT (mg/mL) were determined at which the plasma coagulation time was twice as long as in the control with the addition of 0.05 M Tris-HCl buffer (with 0.175 M NaCl, pH 7.4) instead of sample. When calculating the antithrombin activity (aIIa), NMIC derivatives calibrated the test system for unfractionated heparin (HEPARIN-BELMED, 25,000 units) (RUP Belmedpreparations, Minsk, Belarus) [37].

The effect of NMIC derivatives on one of the links of cellular hemostasis was evaluated based on the results of a platelet aggregation analysis according to the method described by Born [38] using a Model 500 aggregometer (Chrono-Log, Havertown, PA, USA) with a recorder recording changes in the light transmission of platelet-enriched human plasma. A solution of the disodium salt of adenosine-5-diphosphate (ADP) (Sigma-Aldrich, St. Louis, MI, USA) was used as an aggregation inducer. Platelet-rich human plasma (0.450 mL) was incubated (1 min at 37 °C) with NMIC derivatives (0.91–910 µg/mL), then ADP was introduced (final concentration 2 × 10^−5^ M). Plasma with 0.05 M tris-HCl buffer, pH 7.4, containing 0.175 M NaCl was used as a control. Platelet aggregation was determined within 5 min (the light transmission of platelet-depleted human plasma was taken as 100%). The maximum amplitude of the aggregation curve (in%) was determined on the platelet aggregation curve.

The effect of NMIC derivatives on human erythrocyte hemolysis was determined according to the method described by Dash et al. [39]. At the National Medical Research Center for Hematology, Moscow, Russian Federation, the blood of a donor from the ulnar vein was collected into a S-Monovette 5 mL 9NC plastic syringe (Sarstedt AG & Co. KG, Nűmbrecht, Germany). Then, the blood was centrifuged for 15 min at 250 g and erythrocytes were washed three times with a 10 mM phosphate buffer (pH 7.4). Erythrocytes were suspended in a phosphate buffer. Next, 0.5 mL aliquots of NMIC derivatives were placed in test tubes (at concentrations of 2.5–2500 µg/mL) and 0.5 mL of the erythrocyte suspension was added. Distilled water was used as a positive control (k+), and a 10 mM phosphate buffer was used as a negative control (k−). The samples were placed in a thermostat for 2 h at a temperature of 37 °C. After incubation, the samples were centrifuged for 15 min at 250 g. The optical density (OD) of the supernatant was measured at a wavelength of 540 nm using a SmartSpec Plus spectrophotometer (Bio-Rad, CA, USA). Equation (1) was used to calculate the degree of hemolysis (DH%):

DH = (OD_o_ − OD_k−_/OD_k+_ − OD_k−_) × 100% (1)

where ${OD}_{0}$ is the optical density of the solution after incubation with the NMIC derivatives; ${OD}_{k-}$ is the optical density of the solution after incubation with 10 mM phosphate buffer; and ${OD}_{k+}$ is the optical density of the solution after incubation with distilled water.

Statistical analyses of the results and calculations of correlation coefficients were carried out using Microsoft Office Excel 2007 and Statistica 6.0 software packages. The nonparametric Mann–Whitney U test was used to compare abnormally distributed data. The results of the work are presented by arithmetic averages ± standard errors of arithmetic averages from 4–20 independent definitions. Statistically significant differences between the data series were observed at *p* < 0.05.

## 3. Results and Discussion

### 3.1. Synthesis of Chitosan Derivatives

**Figure 1:**
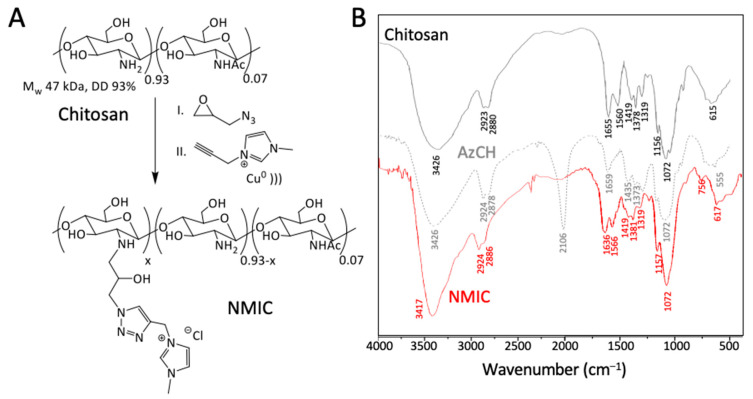
*(***A**)—Synthesis scheme of NMIC chitosan derivatives. (**B**)—FTIR spectra of chitosan and its derivatives.

Copper-catalyzed azide–alkyne cycloaddition (CuAAC) is a simple and effective procedure for covalent bond formation. NMIC 1-5 derivatives were synthesized, as we previously described in [9]. We used low-molecular-weight chitosan as a starting material (Mw 47 kDa, DD 93%). AzCH derivatives were dissolved in acetic acid solution. Copper turnings and the alkyne component were added into the reaction mixture, and then it was sonicated on an ultrasonic bath. The synthesis scheme is presented in Figure 1A. To make sure that the reaction proceeded, we took a small amount (50 µL) of the reaction mixture and added it into a 10% NH_4_OH solution. The azide–alkyne cycloaddition product was completely soluble in the alkaline medium in spite of the starting AzCH in the reaction mixture.

FTIR spectra are presented in Figure 1B. The typical broad peaks of OH and NH stretching vibrations can be observed at 3426 cm^−1^ in all spectra. The appearance of a new stretching vibration −N=N^+^=N^−^ at 2106 cm^−1^ and changes in the NH bending vibrations at 1659 cm^−1^ proved the introduction of a N_3_ group with substitution of the amino groups of chitosan in AzCH. The disappearance of the N_3_ signal at 2106 cm^−1^ in NMIC proved the completion of the reaction [8] and slight changes in the 1636─1319 cm^−1^ region can be seen as a result of CuAAC.

**Figure 2:**
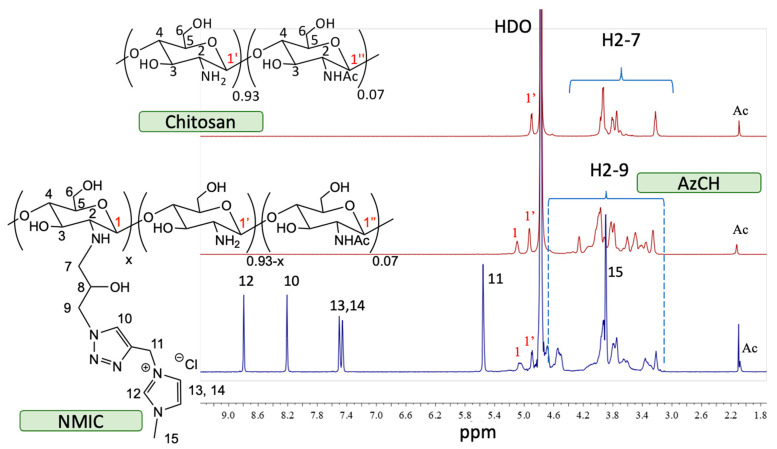
Stack of ^1^H NMR spectra of chitosan/AzCH/NMIC (D_2_O/CF_3_COOH-20/1 50 °C).

The 1H NMR spectra are presented in Figure 2. The starting chitosan material had typical polysaccharide signals (H2-7). Anomeric protons signals H1′ from free glucosamine were presented. After chemical modification, the intensity of H1′ decreased due to the formation of the N-substituted product AzCH. A new signal H1 and additional signals in H2-9 region appeared. The 1H NMR spectrum of NMIC showed new N-methylimidazole (H12 8.79 ppm) and triazole (H10 8.21) signals. Additional signals of −CH=CH− (H13 and H14 at 7.5 and 7.46 ppm) and CH_3_ (H5 at 3.9 ppm) from methylimidazole were observed. A singlet (H11 5.56 ppm) from CH_2_ was also presented.

The characteristics of chitosan derivatives are presented in Table 1.

### 3.2. Solubility of NMIC1-5 Derivatives

**Figure 3:**
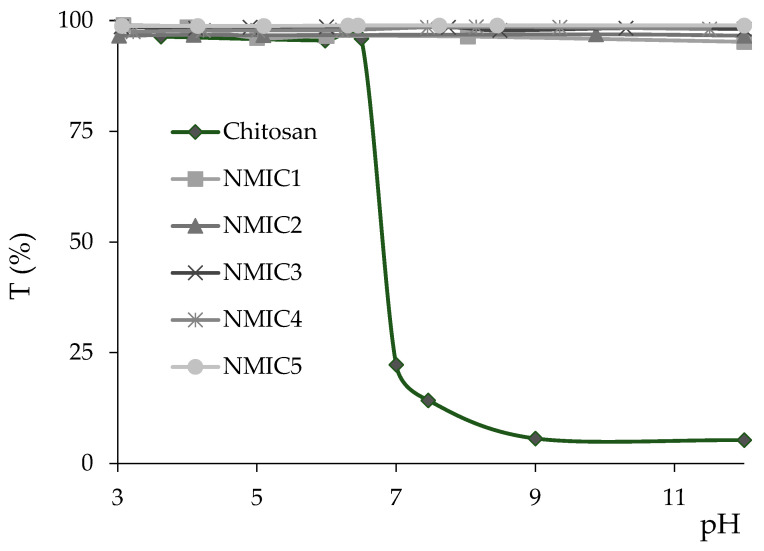
Solubility of NMIC derivatives in the pH 3–12 range.

Introduction of the 3-methylimidazolium fragment greatly increased the solubility of chitosan derivatives. After reaching pH 6.5, chitosan started to precipitate, while all NMIC derivatives were completely soluble in the 3–12 pH range (Figure 3). Introduction of positively charged hydrophilic moieties increased the water solubility of the polymer in comparison to the starting chitosan and azido-derivative. Reaching a DS of 18% is enough for complete solubility in a wide pH range at a 2 mg/mL concentration.

### 3.3. Antibacterial Activity of Chitosan and Its Derivatives

The introduction of a methylimidazole fragment into chitosan increases the antibacterial activity of chitosan derivatives by 4 times compared to the initial chitosan for the studied bacteria. *S. epidermidis* was the most sensitive to the action of chitosan and its NMIC derivatives. However, the activity remained practically unchanged at the degree of substitution from 34% to 76%, which is probably due to the fact that as the amount of the bulk substituent increases it prevents interaction with the bacterial cell wall as compared to the lower DS (18%) [40]. The results are presented in Table 2.

### 3.4. In Vitro Hemocompatibility of NMIC Derivatives

**Figure 4:**
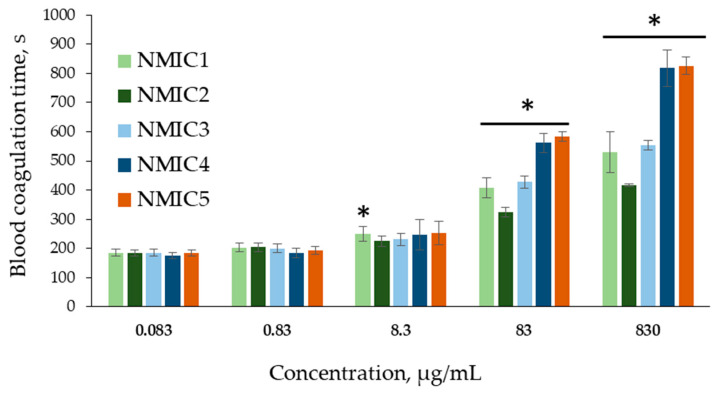
Blood recalcification time (s) during incubation with NMIC derivatives. * *p* < 0.05—the reliability of differences with the indications in the control without adding samples (0 mg/mL; 187.19 ± 10.76 c); n = 6.

To assess the in vitro hemocompatibility of NMIC derivatives, tests were chosen with the help of which it is possible to assess the effect on the coagulation of whole blood and platelet-poor human plasma (BRT, APTT), as well as on the aggregation of human platelets and, indirectly (according to the degree of hemolysis), on the cell membrane of human erythrocytes [41]. Incubation in a blood/plasma test tube of compounds with anticoagulant activity (coagulation factor inhibitors or plasma coagulation factor inhibitors activators) leads to an increase in coagulation time in coagulological tests [21]. To activate clotting, a calcium chloride solution was used in the BRT test, since during blood stabilization, calcium ions necessary for coagulation bind to sodium citrate [35]. The method was developed many years ago to control the level of unfractionated heparin (an activator of plasma inhibitors of coagulation factors) during artificial blood circulation [21]. At concentrations of 0.083 µg/mL and 0.830 µg/mL, all NMIC derivatives and NMIC2, NMIC3, NMIC4 and NMIC5 at concentrations of 8.300 µg/mL did not affect blood coagulation in the BRT test (Figure 4).

Addition of the studied samples to blood at concentrations of 83 µg/mL and 830 µg/mL resulted in a significant increase in the clotting time (324.10 ± 16.45–583.63 ± 15.03 s and 416.78 ± 5.40–825.47 ± 29.71 s, respectively) compared with the control (187.19 ± 10.76 s). That is, starting from a concentration of 83 µg/mL, the samples showed anticoagulant activity. Incubation of blood with NMIC derivatives (degree of substitution 18%, 34%, 48%, 64%, 76%, respectively) at the maximum concentration led to 2.8-, 2.3-, 3.0-, 4.4- and 4.4-fold increases in the blood clotting time, respectively, in comparison with the control. The blood clotting time increased with an increase in the degree of substitution. The correlation coefficients between the degree of substitution and blood clotting time were r 83.000 µg/mL = 0.7304 and r 830.000 µg/mL = 0.7791 (n = 30; *p* = 0). When NMIC samples were added to blood at concentrations of 83 mcg/mL and 830 mcg/mL, a moderate positive relationship was observed between the degree of substitution and the blood clotting time; the correlation coefficients (r) were 0.7304 and 0.7355 (n = 30; *p* = 0), respectively. This means that with an increase in the degree of substitution of NMIC samples, the blood clotting time increased. Unfortunately, we did not find any publications on the association of anticoagulant activity of chitosan derivatives with the degree of substitution by N-methylimidazole. However, in a review Dimassi et al. [42], the authors analyzed data, including data on the the effect of sulfation on the anticoagulant activity of chitosan derivatives. In the article by Yang et al. [43], the authors report that highly sulfated chitosans (degree of substitution >2.1) significantly increase the coagulation time in the APTT test. Vongchan et al. [44] showed that chitosan with a degree of sulfation of 2.13 had a high anticoagulant activity.

**Figure 5:**
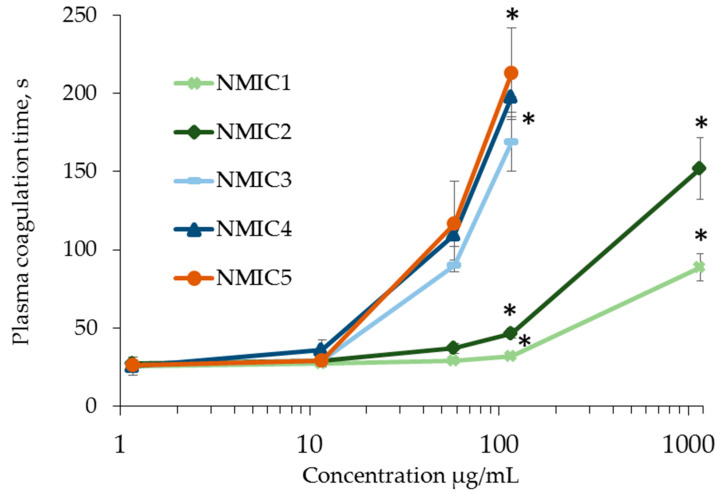
APPT (s) during incubation with NMIC derivatives. * *p* < 0.05—the reliability of differences with the indications in the control without adding samples (0 mg/mL; 30.96 ± 0.89 c); n = 4.

The effect of NMIC derivatives on the coagulation of platelet-poor human plasma during the activation of factor XII by ellagic acid in the presence of soy phospholipids and calcium ions was studied in the APTT test [36]. At concentrations of 1.163 µg/mL and 11.630 µg/mL, the samples did not affect human plasma coagulation. With an increase in the concentration of NMIC derivatives, the plasma coagulation time in the APTT test significantly increased compared with the control (0 µg/mL: 30.96 ± 0.89 c) (Figure 5). The anticoagulant activity of the samples was manifested starting from a concentration of 58.140 µg/mL for NMIC5, with a concentration of 116.280 µg/mL for NMIC2, NMIC3 and NMIC4 and with a concentration of 1162.800 µg/mL for NMIC1. With an increase in the degree of substitution, the plasma coagulation time significantly increased. The correlation coefficient between the degree of substitution and the plasma clotting time was r 116.280 µg/mL = 0.9086 (n = 16; *p* = 0). Yang et al. [43] showed a relationship between the anticoagulant activity of chitosan derivatives and the degree of sulfation. Chitosans with a high degree of sulfation significantly increased the plasma clotting time in the activated partial thromboplastin time test. Obtaining chitosan derivatives with anticoagulant activity is promising for use in the creation of stents, grafts, implants, etc. [45].

The concentrations of the samples at which the plasma coagulation time increased two-fold, compared with the control (2APTT), reached 0.0245 ± 0.0052–0.6950 ± 0.0737 mg/mL (Table 1). The range of antithrombin activity of the samples was 0.0991 ± 0.0099–3.075 ± 0.5866 U/mg (Table 3).

**Figure 6:**
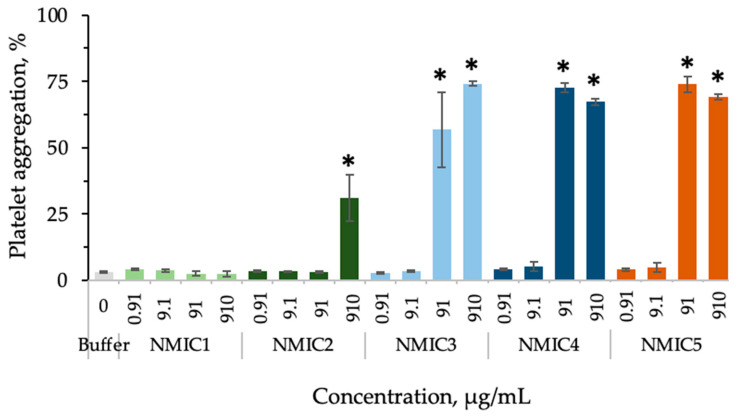
The effect of NMIC derivatives on platelet aggregation. * *p* < 0.05—reliability of differences with indications in the control with a buffer; control with ADP: platelet aggregation 79.23 ± 0.92%; n = 4–15.

The addition of the NMIC1 sample to platelet-rich human plasma with the lowest degree of substitution of 18% (0.910–910 µg/mL) did not affect platelet aggregation (Figure 6). The NMIC2 sample at a concentration of 910 µg/mL provoked platelet aggregation to 31.13 ± 8.76% (in the control 3.19 ± 0.28%). Samples of NMIC3, NMIC4 and NMIC5 did not affect platelet aggregation only at concentrations of 0.910 and 9.100 µg/mL.

**Figure 7:**
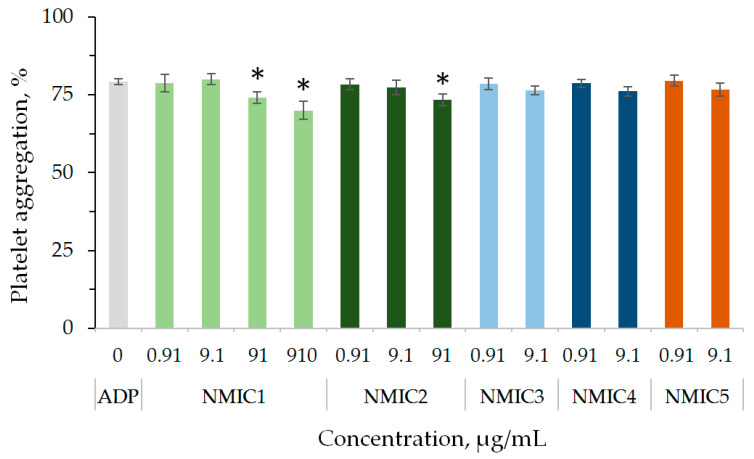
The effect of NMIC derivatives on ADP-induced platelet aggregation. * *p* < 0.05—the reliability of differences with the indications in the control with ADP; n = 4–16.

To analyze the effect on ADP-induced platelet aggregation, concentrations were selected at which no independent effect of samples on platelet aggregation was observed. A slight significant decrease in platelet aggregation in comparison with the control was observed during plasma incubation with NMIC1 and NMIC2 samples at concentrations of 91/910 µg/mL and 91 µg/mL, respectively (Figure 7).

**Figure 8:**
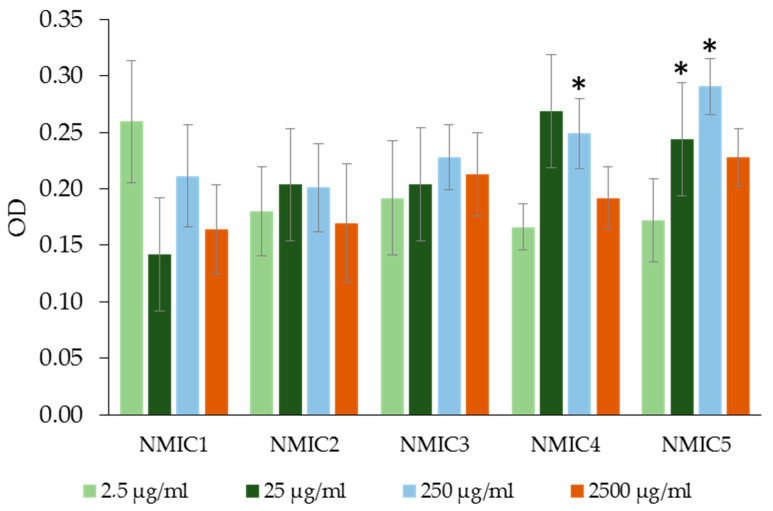
The optical density (OD) of the liquid obtained as a result of centrifugation of an incubation mixture containing washed human erythrocytes with solutions of NMIC derivatives. * *p* < 0.05—the reliability of the differences with the indications in the booster k−; OD k− (with PBS) = −0.16928 ± 0.01601; OD k+ (with H_2_O) = −4 ± 0; n = 5–20.

With an increase in the concentration of samples of NMIC1, NMIC2 and NMIC3 added to the washed human erythrocytes, the optical density of the supernatant obtained by centrifugation after incubation did not differ from the optical density of the buffer control (Figure 8). The degree of hemolysis of erythrocytes during incubation with samples NMIC1, NMIC2 and NMIC3 was 0.174–1.324%. The incubation of washed erythrocytes with sample NMIC4 (250 µg/mL) and sample NMIC5 (25 µg/mL and 250 µg/mL) resulted in an increase in the optical density of the fluid solution by 1.47-, 1.44- and 1.72-fold in comparison with the negative control, respectively. However, the degree of hemolysis for the NMIC4 sample was 0.372 ± 0.364–2.530 ± 1.290%, and for the NMIC5 sample, it was 0.251 ± 0.238–2.943 ± 0.770%. Roy et al. [41] showed that the degree of hemolysis of erythrocytes during incubation with a hydrogel containing imidazole in the structure is 1.07 ± 0.05%. Dennehy et al. [41] showed that two of the three metal complexes containing thiol groups (Zn-tsac) with imidazole bases as co-ligands at a dose of 10 mcg/mL did not provoke the fragility of erythrocyte membranes. Kuthyala et al. [41] observed the absence of the effect of disubstituted imidazo [1,2-a]pyridines on the membranes of human erythrocytes in vitro.

## 4. Conclusions

There is great interest in such biopolymer polysaccharides as cellulose, chitosan, starch, chitin and alginate to create natural substitutes for synthetic polymers [1]. Chitin and its derivatives find practical applications in chemistry, agriculture, medicine and cosmetics, as well as in the textile and paper industry. Recently, chitosan has also aroused great interest, including in the field of biomedicine. Chitosan is a biopolymer derived from chitin which has demonstrated great potential for use in tissue regeneration and controlled drug delivery [14].

We have successfully synthesized new water-soluble chitosan derivatives containing N-methylimidazole moieties (NMIC) using click chemistry. Chemical modification of chitosan increases the solubility with pH and the antibacterial activity compared to original chitosan. In this study, we have shown that adding NMIC samples to a test tube with blood or platelet-poor plasma at concentrations of 83.0 µg/mL or 58.1–1162.8 µg/mL, respectively, leads to an elongation of the coagulation time. The studied compounds have anticoagulant activity, which increases with an increase in the degree of substitution.

The studied NMIC samples with the lowest anticoagulant activity and low degrees of substitution (18% and 34%) at concentrations of up to 91/910 µg/mL did not independently affect platelet aggregation or ADP-induced platelet aggregation, and at concentrations of up to 2500 µg/mL of the erythrocyte suspension, erythrocyte hemolysis was below 1%.

Medical devices/materials in contact with blood are widely used in the diagnosis and treatment of various diseases [21]. Functional coatings of medical devices/materials are promising for obtaining surfaces with a reduced adhesion of plasma proteins and without platelet activation or coagulation cascades, which usually leads to thrombosis in clinical use and poses a serious threat to the health/life of patients. The studied hemocompatible NMIC derivatives are promising for achieving the thromboresistance of the surfaces of materials/devices.

## Figures and Tables

**Table 1:** The degree of substitution of chitosan derivatives.

Derivatives	DS, %
NMIC1	18
NMIC2	34
NMIC3	48
NMIC4	63
NMIC5	76

**Table 2:** Antibacterial activity of chitosan derivatives against *S. epidermidis* and *E. coli*.

Sample	DS, %	MIC (µg/mL)	
		*S. epidermidis*	*E. coli*
Chitosan	**−**	62.5	250
NMIC1	18	7.8	62.5
NMIC2	34	15.6	62.5
NMIC3	48	15.6	62.5
NMIC4	63	15.6	62.5
NMIC5	76	15.6	62.5

**Table 3:** Anticoagulant activity of NMIC derivatives.

Samples	2APTT, mg/mL	Antithrombin Activity, U/mg
Chitosan	0.6950 ± 0.0737	0.0991 ± 0.0099 *
NMIC1	0.2810 ± 0.0442	0.2558 ± 0.0388 *
NMIC2	0.0263 ± 0.0056	2.3105 ± 0.4999
NMIC3	0.0245 ± 0.0052	3.075 ± 0.5866
NMIC4	0.0353 ± 0.0068	2.1321 ± 0.4652
NMIC5	0.2810 ± 0.0442	0.2558 ± 0.0388 *

Note: * *p* < 0.05—the reliability of differences with indications for NMIC4; 2APTT for UFH −0.0667 ± 0.0015 U/mL; n = 4.

## Data Availability

Not applicable.
